# An engineered ligand-responsive Csy4 endoribonuclease controls transgene expression from Sendai virus vectors

**DOI:** 10.1186/s13036-024-00404-9

**Published:** 2024-01-16

**Authors:** Takumi Kishimoto, Ken Nishimura, Kana Morishita, Aya Fukuda, Yusaku Miyamae, Yutaro Kumagai, Kimio Sumaru, Mahito Nakanishi, Koji Hisatake, Masayuki Sano

**Affiliations:** 1 Laboratory of Gene Regulation, Institute of Medicine, University of Tsukuba, 1-1-1 Tennodai, Tsukuba, Ibaraki 305-8575 Japan; 2 Present Address: Department of Clinical Application, Center for iPS Cell Research and Application (CiRA), Kyoto University, 53 Kawahara-cho, Shogoin, Sakyo-ku, Kyoto, 606-8507 Japan; 3 Cellular and Molecular Biotechnology Research Institute, National Institute of Advanced Industrial Science and Technology (AIST), Central 5, 1-1-1 Higashi, Tsukuba, Ibaraki 305-8565 Japan; 4 Institute of Life and Environment Sciences, University of Tsukuba, 1-1-1 Tennodai, Tsukuba, Ibaraki 305-8572 Japan; 5 TOKIWA-Bio, Inc, 2-1-6 Sengen, Tsukuba, Ibaraki 305-0047 Japan

**Keywords:** Sendai virus, Csy4, Ligand, Gene regulation, ES cells, Neural differentiation

## Abstract

**Background:**

Viral vectors are attractive gene delivery vehicles because of their broad tropism, high transduction efficiency, and durable expression. With no risk of integration into the host genome, the vectors developed from RNA viruses such as Sendai virus (SeV) are especially promising. However, RNA-based vectors have limited applicability because they lack a convenient method to control transgene expression by an external inducer.

**Results:**

We engineered a Csy4 switch in Sendai virus-based vectors by combining Csy4 endoribonuclease with mutant FKBP12 (DD: destabilizing domain) that becomes stabilized when a small chemical Shield1 is supplied. In this Shield1-responsive Csy4 (SrC) switch, Shield1 increases Csy4 fused with DD (DD-Csy4), which then cleaves and downregulates the transgene mRNA containing the Csy4 recognition sequence (Csy4RS). Moreover, when Csy4RS is inserted in the viral *L* gene, the SrC switch suppresses replication and transcription of the SeV vector in infected cells in a Shield1-dependent manner, thus enabling complete elimination of the vector from the cells. By temporally controlling BRN4 expression, a BRN4-expressing SeV vector equipped with the SrC switch achieves efficient, stepwise differentiation of embryonic stem cells into neural stem cells, and then into astrocytes.

**Conclusion:**

SeV-based vectors with the SrC switch should find wide applications in stem cell research, regenerative medicine, and gene therapy, especially when precise control of reprogramming factor expression is desirable.

**Supplementary Information:**

The online version contains supplementary material available at 10.1186/s13036-024-00404-9.

## Background

Viral vectors are appealing vehicles of gene delivery for basic research and clinical applications owing to their broad cell tropism, superior transduction efficiency, and durable transgene expression [1, 2]. Currently, in vivo and ex vivo gene delivery employs adeno-associated viral (AAV) or lentiviral (LV) vectors [3, 4] as a primary choice in gene therapy settings. However, these DNA-based vectors have critical disadvantages with regards to long-term durability of transgene expression [5], the maximum size of a packageable transgene [6], or a potential risk of tumor formation by the integrated transgene [7]. Despite extensive studies on other types of DNA-based viral vectors, including adenoviral vectors (AdV) [8], very few DNA-based vectors are available that enable durable expression of a large transgene without the risk of chromosomal integration. Alternatively, other choice of vectors for gene delivery includes RNA-based vectors, which exist in cells as RNA and do not integrate into the host genome [9]. However, RNA-based vectors are incompatible with gene regulatory systems that rely on a DNA-binding transcription factor and a tissue-specific or ligand-inducible promoter. Thus, current RNA-based vectors lack a tunable gene expression system, which should confer significant safety and efficacy in in vivo and ex vivo gene delivery [10–12].

Among the RNA-based vectors, negative-sense single-stranded RNA viruses (NSRVs) have been widely used for oncolytic and vaccine vectors because of their potent cytotoxicity and immunogenicity [13–15]. Besides, NSRVs such as measles virus (MV), vesicular stomatitis virus (VSV), borna disease virus (BDV), and Sendai virus (SeV) have been recognized as a promising vector platform of gene expression in gene and cell therapies as well as in regenerative medicine [16–19]. One of the best studied among NSRV-based vectors is those derived from SeV, which is a member of the *Paramyxoviridae* and possesses a wide-reaching cell and tissue tropism. Its genome consists of a non-segmented negative-strand RNA that encodes six main proteins; nucleocapsid protein (NP), phosphoprotein (P), matrix protein (M), glycoproteins (F and HN), and large protein (L) [20]. Once infected, SeV resides in cytoplasm, where its RNA genome is replicated and transcribed by RNA-dependent RNA polymerase (RdRp) encoded by the *L* gene [20]. An SeV strain Cl.151, originally derived from the cytopathic Nagoya strain, is a temperature-sensitive mutant that shows little cytopathic effects at nonpermissive temperature (38^o^C). At this temperature, Cl.151 fails to produce progeny viruses, but its RNA genome remains persistently in cells and continues to express viral proteins [21]. Importantly, the persistent infection of Cl.151 does not require the structural genes, *M*, *F*, and *HN*, which are used solely for producing progeny virus particles.

Based upon these properties of Cl.151, we previously created a non-transmissive recombinant SeV vector, termed replication-defective and persistent SeV (SeVdp), by completely deleting structural genes, *M*, *F*, and *HN*, from the viral genome [22]. In mammalian cells, the SeVdp vectors remain in cytoplasm, where replication and transcription of the viral vector continue unabated. Thus, transgenes, inserted in place of the *M*, *F*, and *HN* genes, are transcribed and then translated continuously in infected cells. Hence, SeVdp vectors achieve long-term expression of transgenes without chromosomal integration and show little cytopathic effects. Moreover, a single SeVdp vector incorporates multiple genes of a relatively large size, which are driven by viral promoters to achieve high expression. These features are especially suited for cell reprogramming by transcription factors, such as generation of induced pluripotent stem cells (iPSCs), for which durable expression of multiple factors at high levels is imperative [22]. Thus, SeVdp vectors are increasingly utilized as a powerful tool for research in gene therapy, regenerative medicine, and biopharmaceutical development [23–25]. However, because SeVdp vectors exist as a single-stranded RNA in cells, no reliable system is available yet to adjust expression of the transgene from SeVdp in a time- or dose-dependent manner.

In recent years, several attempts have been made to install a tunable gene expression system in NSRV-derived vectors. Although NSRV-derived vectors are not compatible with gene regulation by a DNA-binding transcription factor, they can be equipped with a regulatory system that operates at the RNA or protein level. For example, cell-type specific miRNAs are successfully used to suppress gene expression from RNA vectors that are derived from MV [26, 27], VSV [28] and SeV [29, 30]. Despite its potency, the miRNA-mediated regulatory system is essentially irreversible and totally depends on the selected miRNA, which precludes flexible regulation of gene expression from the vector. Another successful approach employs a ribozyme that cleaves the target mRNA in response to a small-molecule ligand such as theophylline or guanine [31–33]. Ligand-responsive ribozymes regulate the RNA level in a reversible fashion, but their dynamic range is rather limited due to leakage and slow conformational change of ribozymes [34]. Moreover, the insolubility and cytotoxicity of ligands may hamper in vivo application of ligand-responsive ribozymes to NSRV-derived vectors [32].

In addition to the regulatory mechanisms at the RNA level, regulation at the protein level has been applied to NSRV-derived vectors. The protein-level regulation is achieved most conveniently by proteasome-mediated degradation. For instance, Banaszynski et al. engineered an FKBP12 mutant that is degraded constitutively unless a small chemical Shield1 is supplied [35]. When this mutant FKBP12 (DD: destabilizing domain) is fused to a protein, the DD-fused protein expressed in cells is subject to rapid degradation by the proteasome system. Shield1, however, inhibits the function of DD and restores the level of the DD-fused protein in a reversible and dose-dependent manner. Moreover, Bonger et al. reported that an FKBP mutant fused with 19-amino acid degron serves as a ligand-induced degradation (LID) to promote rapid degradation of the LID-tagged protein in a Shield1-dependent manner [36]. Previously, we have successfully used Shield1-responsive KLF4 expressed from SeVdp vectors in somatic cell reprogramming and demonstrated the applicability of the DD-tagged protein in NSRV-derived vectors [37, 38]. Although Shield1-responsive DD serves as a flexible molecular switch, attachment of 108 amino acid as DD tag may alter the structure and function of the target protein, which limits the versatility of the DD tag in clinical applications.

Recent developments of the clustered regularly interspaced short palindromic repeat (CRISPR) system have revolutionized the technologies for DNA-based gene editing and gene regulation, in large part due to Cas9 that can be directed to a specific genomic site by virtue of its cognate guide RNA (gRNA) [39]. More recently, another component of the CRISPR system, Csy4, has gained widespread application in RNA-based technologies. Csy4 is an endoribonuclease that produces gRNAs by processing pre-CRISPR transcript (pre-crRNA) in *Pseudomonas aeruginosa* [40]. Csy4 specifically recognizes the stem-loop structure of the 28-nt conserved sequence in pre-crRNA and efficiently cleaves the 3′ end of the stem region. This sequence-specific cleavage activity of Csy4 has been exploited in genome editing technology [41], design of programmable gene networks [42, 43], analysis of RNA-protein interactions [44] and detection of miRNAs [45].

Here, we integrated Csy4 endoribonuclease with DD to engineer a Shield1-responsive Csy4 (SrC) switch in SeVdp-based vectors. In the cells harboring SeVdp vectors with the SrC switch, Shield1 upregulates the level of Csy4 fused with DD (DD-Csy4), which in turn cleaves the transgene mRNA that contains the Csy4 recognition sequence (Csy4RS), ultimately downregulating transgene expression. Moreover, when Csy4RS is inserted in the viral *L* gene, Shield1 adjusts the amount of the SeVdp vector and, if combined with drug selection, eliminates the vector completely from the infected cells. These features of the SrC switch system are especially suitable for cell reprogramming or directed differentiation by forced expression of a transcription factor.

## Results

### The SrC switch controls gene expression from SeVdp vectors in a Shield1-dependent manner

**Fig. 1:**
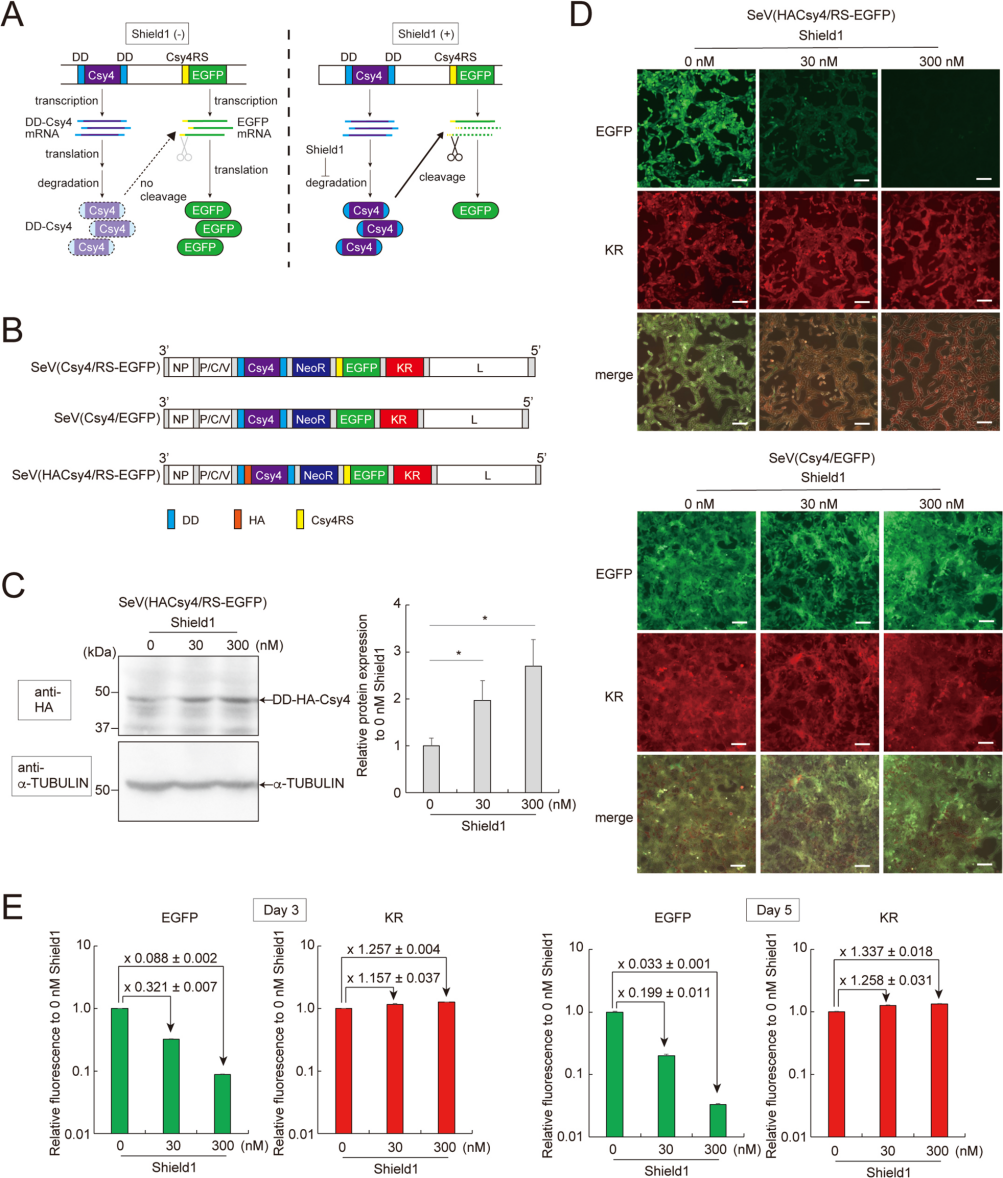
Control of transgene expression from SeVdp vectors using the SrC switch. **A** Outline of the SrC switch system. Stabilizing DD-Csy4 with Shield1 decreases EGFP expression. **B** Structure of SeVdp vectors. NP, P/C/V, and L indicate SeV *NP*, *P/C/V*, and *L* genes, respectively. The *P/C/V* gene contains multiple open reading frames encoding P, C, and V proteins. NeoR: aminoglycoside 3′-phosphotransferase, KR: Keima-Red, DD: destabilizing domain, HA: HA-tag, Csy4RS: Csy4 recognition sequence. **C** DD-Csy4 protein expression upon Shield1 addition. SeV(HACsy4/RS-EGFP)-infected NIH3T3 cells were cultured with the indicated concentration of Shield1 for 3 days. Whole cell lysates extracted from the cells were subjected to Western blotting using anti-HA and anti-α-TUBULIN antibodies. Data are represented as the means ± SEM of three independent experiments. *p < 0.05. **D** Images of EGFP expression controlled by Shield1-responsive Csy4. NIH3T3 cells were infected with SeV(HACsy4/RS-EGFP) or SeV(Csy4/EGFP). After G418 selection, the cells were cultured with the indicated concentration of Shield1 for 5 days. EGFP and KR images were overlaid to produce merged images. Scale bars, 100 μm. **E** Fluorescent protein expression determined via flow cytometry. SeV(Csy4/RS-EGFP)-infected NIH3T3 cells were cultured with the indicated concentration of Shield1. EGFP and KR expression levels were determined using flow cytometry 3 or 5 days after Shield1 addition. Data are represented as the means ± SD of three independent experiments

To equip RNA-based SeVdp vectors with a tunable gene expression system, we employed Csy4 endoribonuclease, which specifically cleaves the RNA harboring the 28-nt Csy4 recognition sequence (Csy4RS) [40]. We also utilized a mutant FKBP12 (DD), which renders DD-tagged protein susceptible to degradation by the ubiquitin-proteasome system [35]. We engineered a gene for Csy4 fused with DD at both the N- and C-termini (DD-Csy4) and the *EGFP* gene containing the Csy4RS at its 5′ untranslated region (5′ UTR). This two-component regulatory system is expected to function in the following way. In the absence of Shield1, DD-Csy4 is degraded rapidly and does not cleave the Csy4RS-containing *EGFP* mRNA, which leads to EGFP expression (Fig. 1A, left). In the presence of Shield1, stabilized DD-Csy4 cleaves Csy4RS-containing *EGFP* mRNA, which diminishes EGFP expression (Fig. 1A, right). We incorporated DD-Csy4 and Csy4RS-containing *EGFP* into an SeVdp vector backbone to create SeV(Csy4/RS-EGFP) (Fig. 1B, top). This vector also encodes Keima-Red (KR) as an internal reference and aminoglycoside 3′-phosphotransferase (NeoR) for selecting infected cells with G418. We also prepared a control vector, SeV(Csy4/EGFP), which carries the *EGFP* gene lacking Csy4RS (Fig. 1B, middle). Additionally, we prepared SeV(HACsy4/RS-EGFP) that carries DD-Csy4 tagged with influenza A virus hemagglutinin (HA) (DD-HA-Csy4) to monitor the expression of DD-Csy4 by using an anti-HA antibody (Fig. 1B, bottom).

First, to test if Shield1 regulates the amount of DD-Csy4 in cells, we infected NIH3T3 cells with SeV(HACsy4/RS-EGFP). Although most of the cells were SeV-positive after infection (Fig. S1), the uninfected cells, if any, were removed by G418 selection to ensure more accurate comparison of the efficacy of SrC switch installed into different SeVdp vectors. The infected cells were then cultured with or without Shield1 for 3 days, and the expression of DD-HA-Csy4 was determined by Western blotting analysis. Addition of 30 and 300 nM Shield1 increased the level of DD-HA-Csy4 by 2.0- and 2.7-fold, respectively (Figs. 1C and S2), demonstrating that Shield1 stabilizes DD-HA-Csy4 and increases its amount in a dose-dependent manner. Next, we tested if Shield1 suppresses expression of the Csy4RS-containing target gene by increasing DD-Csy4. NIH3T3 cells were infected with SeV(Csy4/EGFP) or SeV(HACsy4/RS-EGFP), and the infected cells were selected with G418 before treatment with Shield1 for 5 days. Fluorescence microscopy indicated that addition of 30 and 300 nM Shield1 suppressed EGFP expression in SeV(HACsy4/RS-EGFP)-infected cells, with the suppression more profound at 300 nM (Fig. 1D, top). Expression of KR, an internal reference that lacks Csy4RS in its mRNA, was not affected by Shield1. Likewise, the cells infected with the control vector SeV(Csy4/EGFP), which carries *EGFP* lacking Csy4RS, maintained expression of EGFP even in the presence of Shield1 (Fig. 1D, bottom). These data show that Shield1 stabilizes DD-Csy4 and specifically controls expression of the Csy4RS-containing target gene in a dose-dependent manner.

To quantitatively evaluate the effect of Shield1 addition, we used flow cytometry to measure EGFP and KR expression in SeV(Csy4/RS-EGFP)-infected cells. At day 3 of Shield1 addition, EGFP expression decreased to 32% and 8% in the presence of 30 and 300 nM Shield1, respectively (Fig. 1E, left). When Shield1 treatment was extended to 5 days, EGFP expression further decreased to 19% and 3% in the presence of 30 and 300 nM Shield1, respectively (Fig. 1E, right). Under all these conditions, KR expression remained essentially unchanged regardless of the presence of Shield1 (Fig. 1E). These quantitative data are consistent with cell images obtained by fluorescence microscopy (Fig. S3).

**Fig. 2:**
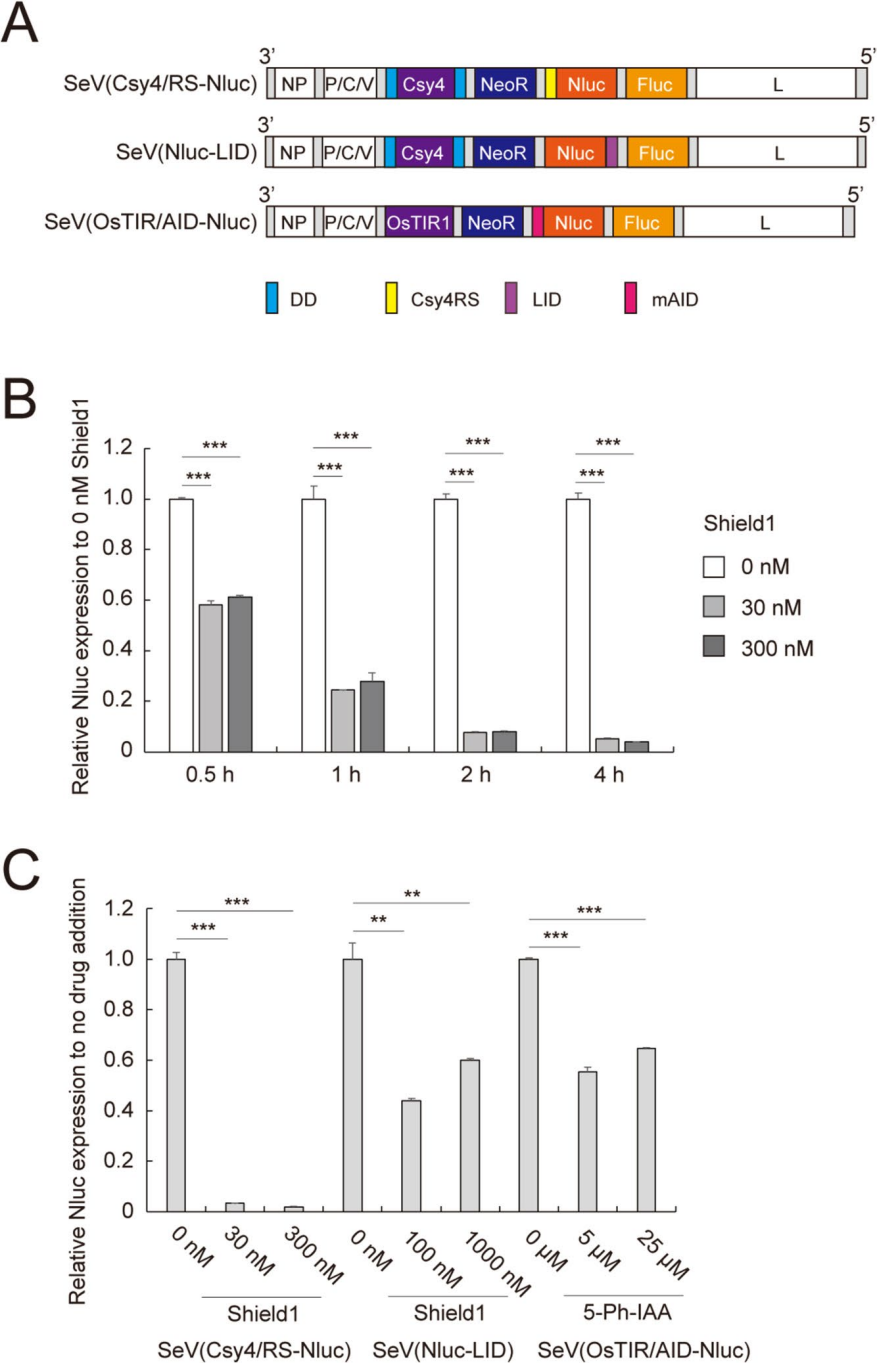
Evaluation of the SrC switch to control luciferase expression.** A** Structure of SeVdp vectors containing various degrons. Nluc: NanoLuc-PEST, Fluc: firefly luciferase (Luc2CP), OsTIR1: *Oryza sativa* F-box transport inhibitor response 1 mutant [OsTIR1(F74G)], LID: ligand-induced degron, mAID: mini auxin-inducible degron. **B** Inhibition of luciferase expression by Shield1-responsive Csy4. SeV(Csy4/RS-Nluc)-infected NIH3T3 cells were treated with 30 or 300 nM Shield1 for indicated time, and then Nluc and Fluc activities were determined, followed by calculating Nluc/Fluc values. Data are represented as the means ± SD of three independent experiments. ***p < 0.001. **C** Comparison of different degron systems. SeV(Csy4/RS-Nluc)- and SeV(Nluc-LID)-infected NIH3T3 cells were treated with Shield1 at indicated concentrations. SeV(OsTIR/AID-Nluc)-infected NIH3T3 cells were treated with 5-Ph-IAA at indicated concentrations. Twenty-four hours after addition of drugs, Nluc and Fluc activities were determined, followed by calculating Nluc/Fluc values. Data are represented as the means ± SD of three independent experiments. **p < 0.01, ***p < 0.001

For more quantitative analysis, we performed a luciferase-based chemiluminescence assay. We prepared SeV(Csy4/RS-Nluc) that carries the genes for DD-Csy4, NeoR, Csy4RS-containing NanoLuc (Nluc) and firefly luciferase (Fluc) (Fig. 2A, top). In this system, Nluc expression should be inhibited by DD-Csy4 with Shield1 whereas Fluc expression remains unchanged. Thus, the value of Nluc versus Fluc activities indicates normalized expression level of Nluc. We infected NIH3T3 cells with SeV(Csy4/RS-Nluc) and selected the infected cells with G418. Then, the infected cells were treated with 30 and 300 nM Shield1 for 0.5, 1, 2, and 4 h, and the Nluc and Fluc activities were determined. The Nluc activity was significantly reduced 0.5 h after addition of Shield1 and gradually decreased with further incubation (Fig. 2B). Notably, 4 h later, Nluc activities was reduced by 95% and 96% in the presence of 30 and 300 nM Shield1, respectively (Fig. 2B). These data suggest that DD-Csy4 is quickly stabilized with Shield1 and immediately cleaves a target mRNA. In addition, 30 nM Shield1 was sufficient to regulate the Nluc expression by the SrC switch presumably because of the short half-life of the target protein.

Next, we compared the potency of SrC switch with that of different protein degradation systems including ligand-induced degron (LID) and auxin-inducible degron version 2 (AID2) systems. The LID system leads to degradation of a target protein fused with an FKBP mutant and 19 amino acid degron in Shield1-dependent manner [36]. On the other hand, the AID2 system facilitates degradation of a target protein fused with 7 kD degron derived from *Arabidopsis* IAA17 (mini(m)AID) in the presence of *Oryza sativa* F-box transport inhibitor response 1 mutant [OsTIR1(F74G)] and 5-Ph-IAA [46]. We incorporated either LID or AID2 components into SeVdp vectors, yielding SeV(Nluc-LID) or SeV(OsTIR/AID-Nluc) (Fig. 2A). Both systems used Nluc and Fluc as an indicator and internal reference, respectively. NIH3T3 cells were infected with SeV(Nluc-LID) and SeV(OsTIR/AID-Nluc) as well as SeV(Csy4/RS-Nluc), and luciferase activities were determined 24 h after addition of drugs. Although SeV(Nluc-LID) and SeV(OsTIR/AID-Nluc) exhibited modest inhibitory effect on the expression of target protein responding to Shield1 and 5-Ph-IAA, respectively, SeV(Csy4/RS-Nluc) strongly inhibited the target expression depending on Shield1 (Fig. 2C), suggesting significant potency of SrC switch when incorporated into the SeVdp vector.

### Reversible and bi-directional control of SeVdp-mediated transgene expression using the SrC switch

**Fig. 3:**
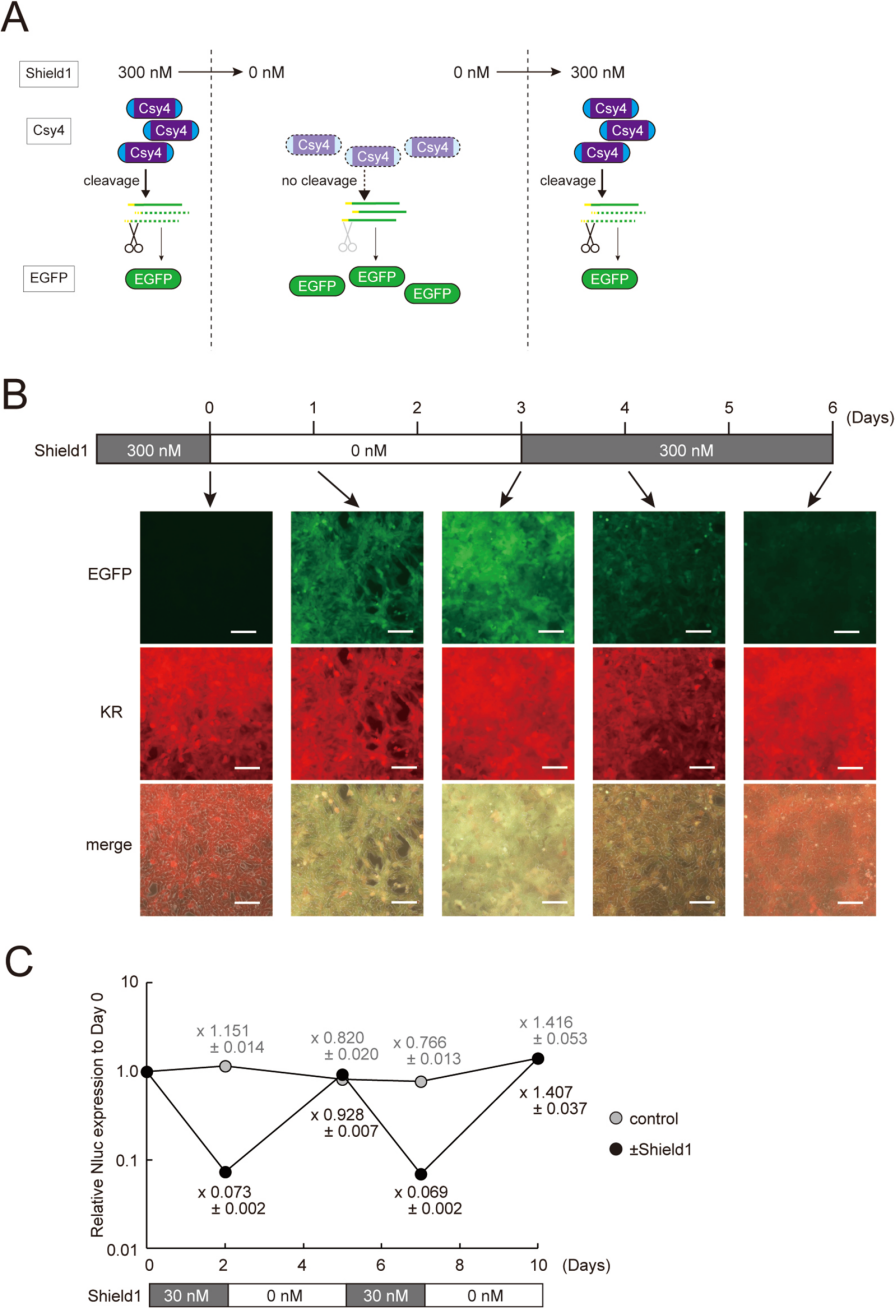
Reversible control of transgene expression by Shield1-responsive Csy4. **A** Outline of reversible control of EGFP expression in the SrC switch system. **B** Images of fluorescent protein expression with altered Shield1 concentration. The concentration of Shield1 was altered as indicated in the scheme to control transgene expression in NIH3T3 cells harboring SeV(Csy4/RS-EGFP). Scale bars, 100 μm. **C** Expression of luciferase gene with altered Shield1 concentration. Addition and removal of Shield1 (± Shield1) or DMSO (control) were repeated as indicated periods in SeV(Csy4/RS-Nluc)-infected NIH3T3 cells. The Nluc and Fluc activities were determined, and Nluc/Fluc ratio in non-treated cells (Day 0) was set to 1.0. Data are represented as the means ± SD of three independent experiments

We sought to confirm if the SrC switch controls transgene expression in a reversible fashion. To this end, we first treated NIH3T3 cells harboring SeV(Csy4/RS-EGFP) (Fig. 1B, top) with 300 nM Shield1 for 5 days, and then Shield1 was removed from culture medium (Fig. 3A). As shown in Fig. 3B, EGFP expression increased only one day after removal of Shield1 and fully recovered after two more days of cell culture without Shield1. When Shield1 was added back to culture medium, however, EGFP expression decreased considerably at day 4 and appeared to be almost completely suppressed at day 6 (Fig. 3B). For quantitative analysis, we examined the Nluc expression in NIH3T3 cells harboring SeV(Csy4/RS-Nluc) (Fig. 2A, top). Addition of 30 nM Shield1 significantly lowered the Nluc activity, but removal of Shield1 fully recovered its activity (Fig. 3C). These data indicate that the SrC switch reversibly controls transgene expression from the SeVdp vector.

We next tried bi-directional control of transgene expression from the SeVdp vector by Shield1 treatment. The DD was fused to the N-terminus of EGFP, and Csy4RS was inserted into the 5′ UTR of *KR* in SeV(Csy4/EGFP) to yield SeV(Csy4/DD-EGFP/RS-KR) (Fig. S4A). In the absence of Shield1, SeV(Csy4/DD-EGFP/RS-KR)-infected NIH3T3 cells expressed KR with markedly reduced EGFP expression because of DD-mediated degradation of EGFP and Csy4 simultaneously. In contrast, addition of 300 nM Shield1 led to upregulation of EGFP and suppression of KR via stabilization of DD-tagged EGFP and DD-Csy4, respectively (Fig. S4B). This data suggests that the SrC switch enables robust and bi-directional control of transgene expression derived from single SeVdp vector backbone by combining with conventional DD tagging strategy.

### The SrC switch controls transgene expression via suppression of L protein expression of the SeVdp vector

**Fig. 4:**
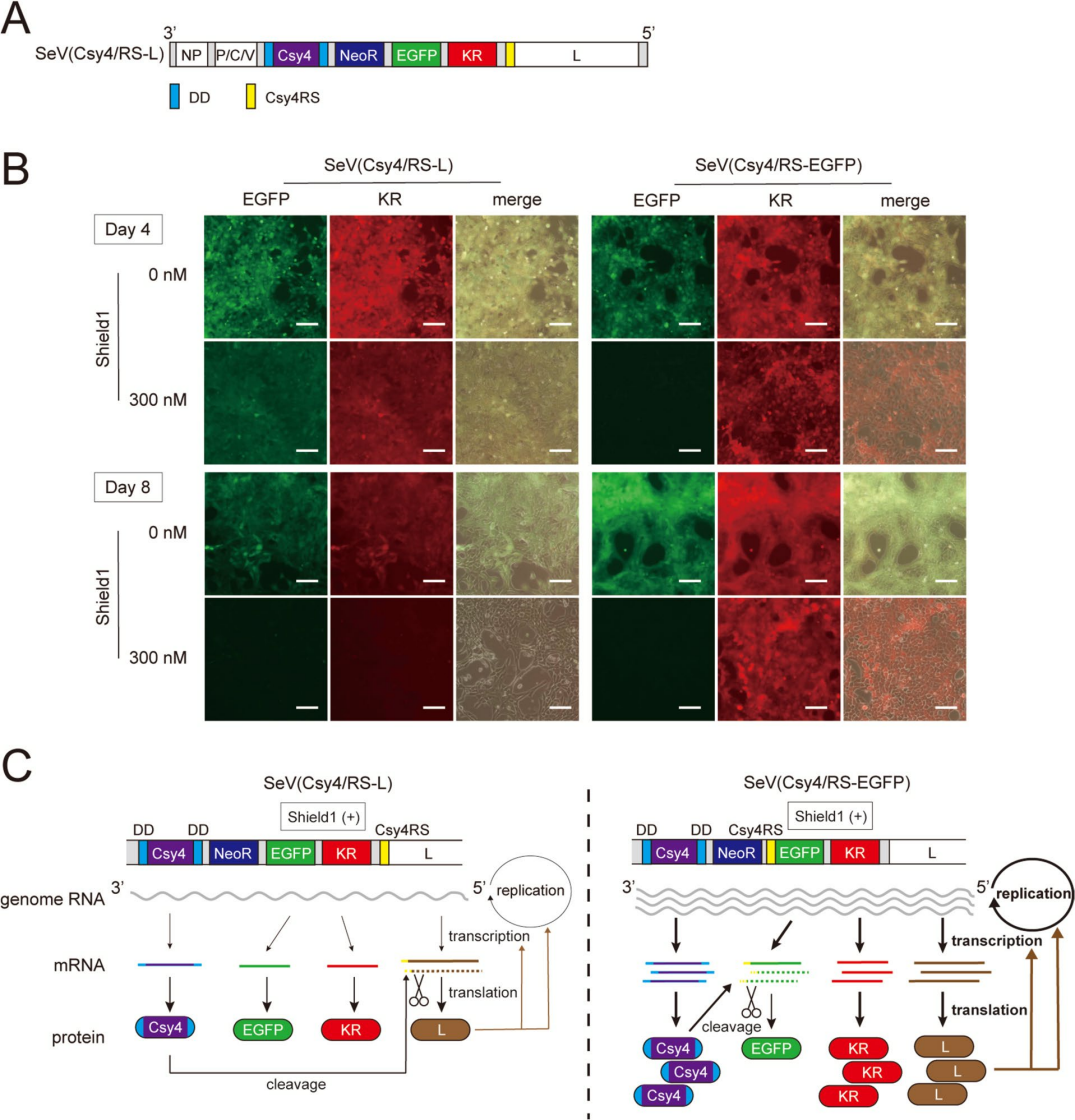
Reduction of transgene expression by DD-Cys4 targeting to the SeV *L *gene.** A** Structure of the SeV(Csy4/RS-L) vector. **B** Images of fluorescent protein expression from SeVdp vectors whose replication and transcription were suppressed by Shield1-responsive Csy4. NIH3T3 cells were infected with SeV(Csy4/RS-L) or SeV(Csy4/RS-EGFP). After G418 selection, the cells were cultured with or without 300 nM Shield1 for 4 or 8 days. Scale bars, 100 μm. **C** Outline of transcription and replication control in the SrC switch system

SeV has a single-stranded negative-strand RNA as a genome, and the virus replicates and transcribes its genome by the RNA-dependent RNA polymerase (RdRp) complex consisting of L and P proteins [47]. We previously reported that siRNA against the *L* gene reduces the transgene expression from SeVdp-based vectors due to impaired SeVdp RNA synthesis [22]. To examine if the SrC switch reduces transgene expression through suppression of *L* gene expression, we prepared an SeVdp vector SeV(Csy4/RS-L) that carries Csy4RS at the 5′ UTR of the *L* gene (Fig. 4A) and, as control, SeV(Csy4/RS-EGFP) that carries Csy4RS at the 5′ UTR of *EGFP* (Fig. 1B, top). When treated with 300 nM Shield1 for 4 days, SeV(Csy4/RS-L)-infected cells decreased EGFP expression moderately whereas SeV(Csy4/RS-EGFP)-infected cells decreased EGFP expression considerably (Fig. 4B). However, after treatment with 300 nM Shield1 for 8 days, SeV(Csy4/RS-L)-infected cells decreased EGFP expression markedly, almost to a similar degree by SeV(Csy4/RS-EGFP)-infected cells (Fig. 4B). Importantly, KR and EGFP expression in SeV(Csy4/RS-L)-infected cells were decreased comparably, indicating that lower EGFP expression is due to the reduced SeVdp vector genome caused via suppression of the *L* gene by the Csy4 switch (Fig. 4C, left). By contrast, even when EGFP expression decreased, KR expression in SeV(Csy4/RS-EGFP)-infected cells remained almost unchanged, indicating that the EGFP expression is solely due to the reduced *EGFP* mRNA caused by the Csy4 switch (Fig. 4C, right). Together, these data show that the *L*-targeting SrC switch modulates the entire transgene expression from the SeVdp vector; however, the *L*-targeting SrC switch requires a prolonged Shield1 treatment to show an effect as compared with the SrC switch that directly targets the transgene mRNA.

### The SrC switch eliminates SeVdp vectors from infected cells by downregulating *L *gene expression

**Fig. 5:**
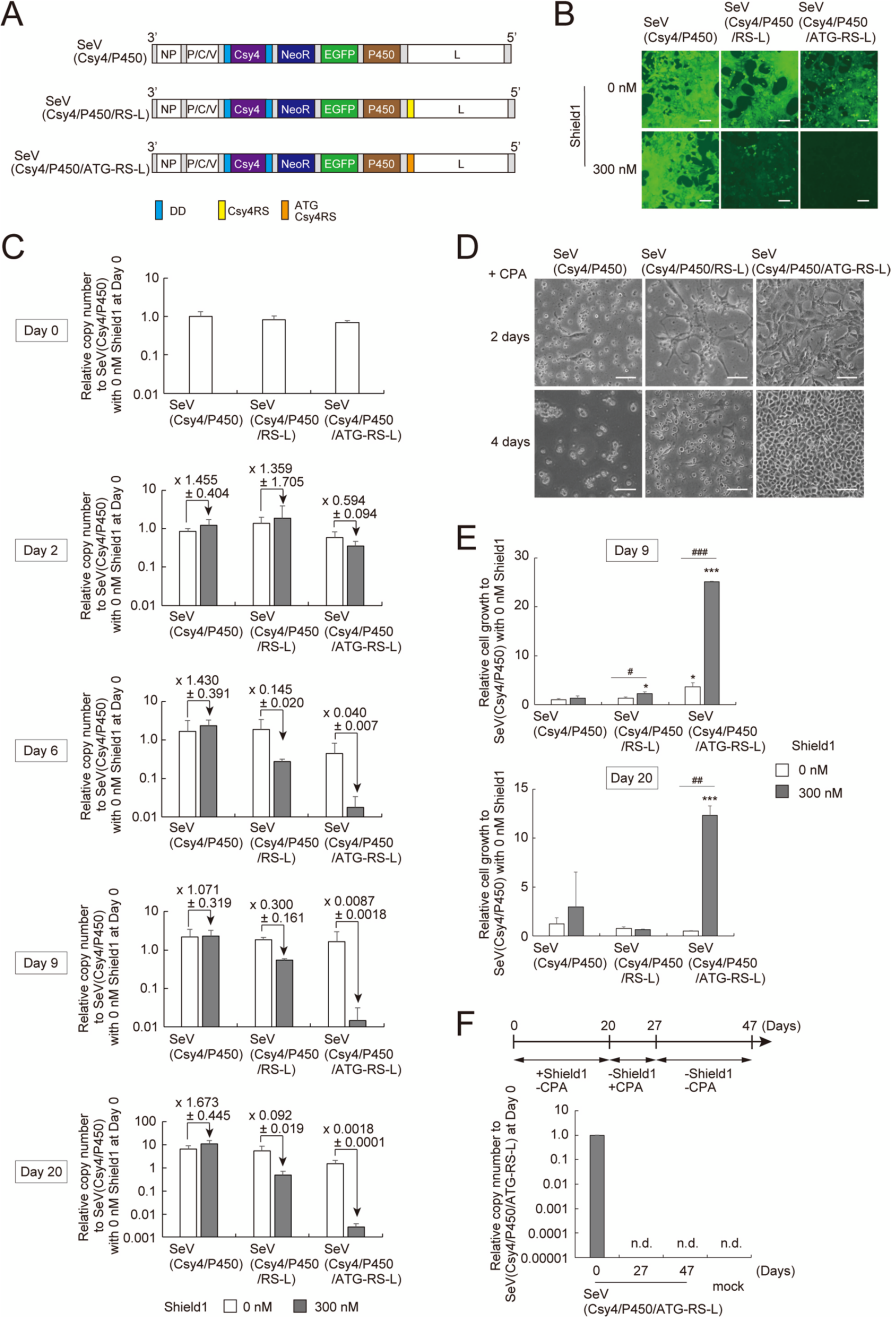
Reduction of the copy number of SeVdp genome by Shield1-responsive Csy4.** A** Structure of SeVdp vectors. P450: rat cytochrome P450 2B1, ATG Csy4RS: Csy4 recognition sequence inserted immediately after the start codon of the *L* gene. **B** Inhibition of viral replication and transcription to change EGFP expression. NIH3T3 cells were infected with the indicated vector and selected with G418. The vector-infected cells were cultured with or without 300 nM Shield1 for 6 days. Scale bars, 100 μm. **C** Relative copy numbers of the vector genome after inhibition of viral replication and transcription. Cells prepared as **B** were cultured with or without 300 nM Shield1. At indicated date, RNA was extracted from the cells, and the amount of SeVdp genomic RNA was determined by RT-qPCR. Data are represented as the means ± SEM of three independent experiments. **D** Selection of SeVdp vector-free cells using P450-based negative selection. NIH3T3 cells infected with the indicated vector were cultured with 300 nM Shield1 for 20 days to eliminate the vector from the cells, and then residual vector-harboring cells were removed by exposure with 1 mM CPA for 2 or 4 days. Scale bars, 100 μm. **E** Crystal violet assay of vector-free cells. NIH3T3 cells infected with the indicated vector were cultured with or without 300 nM Shield1 for 9 or 20 days, followed by exposure with 1 mM CPA for 7 days. The cells were subjected to Crystal violet assay. Data are represented as the means ± SEM of three independent experiments. *p < 0.05 and ***p < 0.001 versus SeV(Csy4/P450)-infected cells cultured without Shield1. ^#^*p* < 0.05. ^##^*p* < 0.01. ^###^*p* < 0.001. **F** Determination of the relative copy numbers of the vector genome in cells after Shield1 and CPA treatments. NIH3T3 cells infected with SeV(Csy4/P450/ATG-RS-L) were cultured with 300 nM Shield1 for 20 days and then with 1 mM CPA for additional 7 days (27 days) followed by cell culture without Shield1 and CPA for 20 days (47 days). The copy number of SeV genomic RNA in the cells was determined by RT-qPCR. Data are represented as the means ± SEM of three independent experiments. mock: uninfected cells, n.d: not detected

We previously showed that prolonged suppression of *L* gene expression may eliminate SeVdp vectors from the infected cells [22, 48]. If the SeVdp vector can be eliminated completely, it becomes possible to generate iPSCs or differentiated cells that are free of the vector once the transcription factor-mediated reprogramming is accomplished. Such vector-free reprogrammed cells have great potential in clinical applications [20]. To investigate if the *L*-targeting SrC switch eliminates SeVdp vectors from the infected cells in a controlled manner, we prepared SeVdp vectors, SeV(Csy4/P450), SeV(Csy4/P450/RS-L) and SeV(Csy4/P450/ATG-RS-L). All three vectors encode DD-Csy4, NeoR, EGFP and rat cytochrome P450 2B1 (P450) (Fig. 5A). SeV(Csy4/P450) lacks Csy4RS and is a control vector. SeV(Csy4/P450/RS-L) carries Csy4RS at the 5′ UTR of the *L* gene, and SeV(Csy4/P450/ATG-RS-L) carries Csy4RS immediately after the start codon of the *L* gene. Both vectors are expected to turn off the *L* gene expression upon Shield1 addition. To assess the presence of SeVdp vector in the infected cells as well as to eliminate the SeVdp vector completely from the infected cells, we added the cytochrome P450 gene to the vector. Cytochrome P450 catalyzes cyclophosphamide (CPA) into the toxic phosphoramide mustard that efficiently kills cells [49, 50]. We infected NIH3T3 cells with each SeVdp vector and removed uninfected cells with G418. Six days after treatment with 300 nM Shield1, SeV(Csy4/P450/RS-L)- and SeV(Csy4/P450/ATG-RS-L)-infected cells decreased EGFP expression (Fig. 5B). Notably, the degree of reduction in EGFP expression was more pronounced in the cells infected with SeV(Csy4/P450/ATG-RS-L) than those with SeV(Csy4/P450/RS-L). Thus, DD-Csy4 cleaves Csy4RS placed after the start codon of the *L* gene more efficiently, which agrees with a previous report [51].

We then quantified the amount of the SeVdp genomic RNA by quantitative reverse transcription-PCR (RT-qPCR). SeV(Csy4/P450)-, SeV(Csy4/P450/RS-L)-, and SeV(Csy4/P450/ATG-RS-L)-infected cells showed similar levels of SeVdp genomic RNA at day 0 (Fig. 5C, top). Upon treatment with 300 nM Shield1 for 9 or 20 days, the amounts of the SeVdp genomic RNA reduced by 70.0% or 90.8% for SeV(Csy4/P450/RS-L) and by 99.13% or 99.82% for SeV(Csy4/P450/ATG-RS-L) (Fig. 5C). Consistent with the data in Fig. 5B, the vector RNA was reduced more sharply in SeV(Csy4/P450/ATG-RS-L)-infected cells than SeV(Csy4/P450/RS-L)-infected cells. These results suggest that the SrC switch suppresses the replication of SeVdp genome by targeting the *L* gene. Besides, Csy4RS placed after the start codon of the *L* gene reduces the amount of the SeVdp genome more sharply in response to DD-Csy4 stabilized by Shield1.

Given the efficient reduction of the SeVdp genome by the SrC switch, we wished to determine what percentage of cells have eliminated the SeVdp vector. Additionally, we tested the feasibility of CPA selection to obtain a population of cells that are entirely free of the vector. We infected NIH3T3 cells with SeV(Csy4/P450), SeV(Csy4/P450/RS-L), and SeV(Csy4/P450/ATG-RS-L) and removed uninfected cells with G418 selection. The cells were cultured for 20 days in the presence of 300 nM Shield1 to reduce the SeVdp genome, and then additionally treated with 1 mM CPA for 2 or 4 days. As expected, SeV(Csy4/P450)-infected cells were killed noticeably (Fig. 5D, left panels), indicating that most of the cells harbored SeVdp genome expressing cytochrome P450. Somewhat unexpectedly, SeV(Csy4/P450/RS-L)-infected cells poorly survived after the CPA treatment (Fig. 5D, middle panels), suggesting that most of the cells still retained the vector despite its amount per cell may have decreased markedly upon Shield1 treatment. By contrast, SeV(Csy4/P450/ATG-RS-L)-infected cells survived quite well after the CPA treatment, which suggests that most of the cells no longer harbored the vector (Fig. 5D, right panels).

To quantitively corroborate these results, we cultured the SeV(Csy4/P450)-, SeV(Csy4/P450/RS-L)-, or SeV(Csy4/P450/ATG-RS-L)-infected cells in the presence of 300 nM Shield1 for 9 or 20 days and subsequently treated the cells with CPA for 7 days. The relative numbers of vector-free cells were determined by staining with Crystal violet (Fig. S5), followed by measurement of absorbance at 570 nm. SeV(Csy4/P450/ATG-RS-L)-infected cells that were treated with Shield1 exhibited a higher value of absorbance as compared with the cells infected with other vectors (Fig. 5E), suggesting that the SrC switch eliminates the SeVdp vector most efficiently in the SeV(Csy4/P450/ATG-RS-L)-infected cells. We further examined the copy number of SeVdp genomic RNA in the CPA-resistant cells using RT-qPCR. When SeV(Csy4/P450/ATG-RS-L)-infected cells were treated with Shield1 for 20 days, the cells still harbored a small amount of the SeVdp vector (Fig. 5C). After CPA treatment for 7 days, however, the vector RNA isolated from the cells was reduced below the detection limit (Fig. 5F; Day 27). Importantly, even in the cells cultured without Shield1 for additional 20 days, vector RNA did not recover from the undetectable level (Fig. 5F; Day 47). These data indicate that the SrC switch that targets the *L* gene virtually eliminates the SeVdp vector from infected cells, and further CPA selection thereafter removes any residual cells that may still harbor the vector. Thus, the SrC switch combined with CPA selection may be applicable for obtaining the vector-free cells that expressed transgenes for the defined period.

### The SrC switch controls BRN4-directed reprogramming of mESCs to neural cells

Because the SrC switch reversibly controls transgene expression for the SeVdp vector, we wished to know if the SrC switch is applicable to direct reprogramming mediated by a transcription factor. To this end, we chose neural cell differentiation from embryonic stem cells (ESCs) as a model system and focused on BRN4, a POU domain transcription factor that plays important roles in development of the nervous system [52]. BRN4 facilitates direct reprogramming of fibroblasts to neural stem cells (NSCs) together with SOX2, KLF4, and c-MYC [53]. We therefore expected that controlling the expression of exogenous BRN4 may enhance neural differentiation of mouse ESCs (mESCs).

**Fig. 6:**
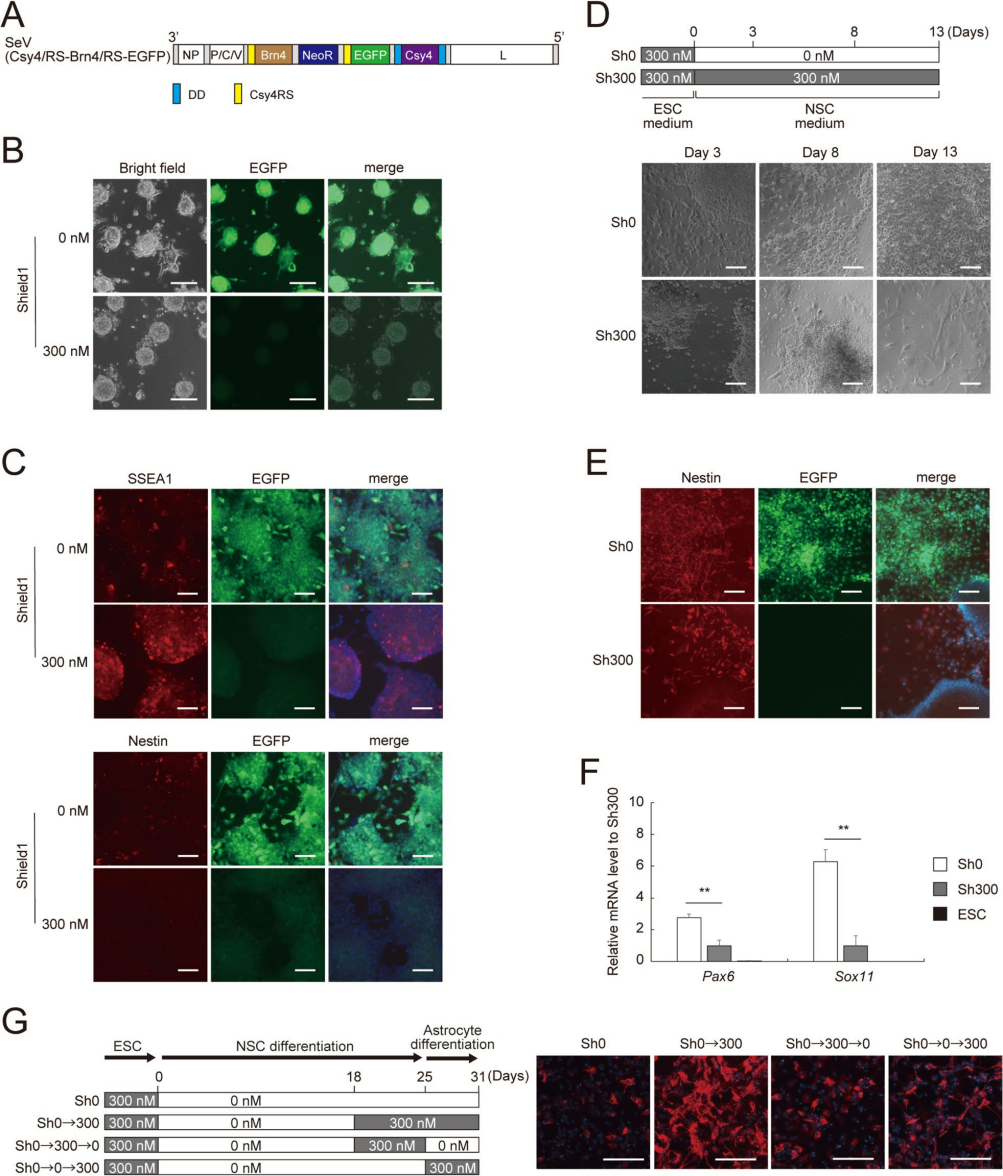
Neural differentiation of mESCs by SrC switch-controlled expression of BRN4.** A** Structure of the SeV(Csy4/RS-Brn4/RS-EGFP) vector. **B** Morphology of mESCs harboring SeV(Csy4/RS-Brn4/RS-EGFP). SeV(Csy4/RS-Brn4/RS-EGFP)-infected EB5 cells were selected with G418 in the presence or absence of 300 nM Shield1. Morphology and EGFP expression were observed 5 days after infection. Scale bars, 100 μm. **C** SSEA1 and Nestin expression in mESCs. SeV(Csy4/RS-Brn4/RS-EGFP)-infected EB5 cells were prepared as described in **B**. SSEA1 and Nestin were immunostained 7 days after the vector infection. Scale bars, 50 μm. **D** Morphology of differentiated NSCs. SeV(Csy4/RS-Brn4/RS-EGFP)-infected EB5 cells were differentiated into NSCs using NSC medium. Shield1 concentration was changed according to the indicated scheme. Morphology of the cells were observed at days 3, 8, and 13. Scale bars, 100 μm. **E** Nestin expression in differentiated NSCs. SeV(Csy4/RS-Brn4/RS-EGFP)-infected EB5 cells were differentiated into NSCs as **D**. Nestin was immunostained at day 7 of the NSC differentiation. Scale bars, 100 μm. **F** NSC marker expression in differentiated NSCs. SeV(Csy4/RS-Brn4/RS-EGFP)-infected EB5 cells were differentiated into NSCs as described in **D**. *Pax6* and *Sox11* mRNA levels in the cells were determined at day 15 of the NSC differentiation. Data are represented as the means ± SEM of three independent experiments. **p < 0.01. **G** GFAP expression in differentiated astrocytes. NSCs differentiated from SeV(Csy4/RS-Brn4/RS-EGFP)-infected EB5 cells were cultured under the indicated condition before and after astrocyte differentiation. GFAP was immunostained at day 6 of the astrocyte differentiation. Scale bars, 100 μm

We prepared the vector SeV(Csy4/RS-Brn4/RS-EGFP) encoding BRN4, NeoR, EGFP, and DD-Csy4 (Fig. 6A). Csy4RS was inserted into the 5′ UTRs of both *Brn4* and *EGFP* genes so that DD-Csy4 regulates expression of BRN4 and EGFP in parallel. We then infected mESCs (EB5 cells) with SeV(Csy4/RS-Brn4/RS-EGFP) and cultured the cells for 5 days in mESC medium containing G418 in the presence or absence of 300 nM Shield1. In the presence of Shield1, the cells lowered EGFP expression and formed dome-like colonies typically observed for mESCs (Fig. 6B, lower panels), due to reduced BRN4 expression (Fig. S6). In the absence of Shield1, however, the cells continued to express EGFP and formed flattened colonies in which some cells spread outward from the periphery of the colonies (Fig. 6B, upper panels), suggesting that high BRN4 expression triggered mESCs to exit from pluripotency toward differentiation. To confirm the effect of BRN4 expression on mESCs, we analyzed expression of ESC marker SSEA1 and NSC marker Nestin in SeV(Csy4/RS-Brn4/RS-EGFP)-infected mESCs. The Shield1-treated cells expressed SSEA1 but not Nestin whereas the untreated cells showed lower SSEA1 expression and higher Nestin expression (Fig. 6C). These data suggest that the SrC switch controls BRN4 expression, which impacts transition of mESCs from pluripotency to neural differentiation.

Given that BRN4 induces mESCs to exit from pluripotency toward neural differentiation, we combined standard neural differentiation medium with the BRN4-expressing SeVdp to devise a protocol for more efficient neural differentiation. We first infected mESCs with SeV(Csy4/RS-Brn4/RS-EGFP) and selected the vector-harboring cells with G418. At this stage, BRN4 expression was suppressed by 300 nM Shield1 to prevent premature differentiation of the mESCs. Then, the vector-harboring mESCs were allowed to differentiate in NSC differentiation medium for 13 days in the presence or absence of Shield1 (Fig. 6D). Both Shield1-treated and -untreated cells differentiated into NSCs; however, the Shield1-untreated cells produced a larger number of Nestin-positive cells, indicating that exogenous BRN4 has an additional promoting effect on NSC differentiation in the standard NSC differentiation medium (Fig. 6D and E). To further corroborate the effect of exogenous BRN4, we analyzed NSC markers, *Pax6* and *Sox11* by RT-qPCR. As shown in Fig. 6F, expression of *Pax6* and *Sox11* was robustly increased when BRN4 expression was induced by removal of Shield1, confirming the effect of BRN4 to promote neural differentiation of mESCs.

Finally, we sought to test if flexible control of BRN4 expression enhances differentiation of mESCs into astrocytes. Following the procedure described in Fig. 6D, we first generated NSCs from mESCs by culturing SeV(Csy4/RS-Brn4/RS-EGFP)-infected mESCs in mESC medium with 300 nM Shield1 and then in NSC differentiation medium without Shield1 (Fig. 6G). At day 25, the medium was replaced by the astrocyte differentiation medium, and expression of the astrocyte marker GFAP was analyzed by immunofluorescence at day 31. After day 18 of the differentiation procedure, we varied the timing of Shield1 addition to change BRN4 expression, as outlined in Fig. 6G. GFAP immunofluorescence showed that SeV(Csy4/RS-Brn4/RS-EGFP)-infected cells differentiated poorly into astrocytes in the absence of Shield1 (Fig. 6G; Sh0), suggesting that continuous expression of BRN4 deters NSCs from differentiating into astrocytes. By contrast, when Shield1 addition was initiated 7 days before astrocyte differentiation (day 18) to downregulate BRN4, NSCs differentiated efficiently into astrocytes (Fig. 6G; Sh0→300). To further dissect the required timing of BRN4 downregulation, Shieid1 was added either before (day 18 to day 25) or after (day 25 to day 31) the start of astrocyte differentiation (Fig. 6G). Addition of Shield1 for 7 days before the start of astrocyte differentiation increased GFAP fluorescence only slightly (Fig. 6G; Sh0→300→0). Likewise, addition of Shield1 for 7 days after the start of astrocyte differentiation increased GFAP fluorescence only moderately (Fig. 6G; Sh0→0→300). These results indicate that BRN4 downregulation should be initiated before the start of astrocyte differentiation and maintained thereafter to permit NSCs to differentiate efficiently into astrocytes. Together, the SrC switch maximizes the efficiency of astrocyte differentiation by flexible tuning of exogenous BRN4 expression to meet the varying requirements for BRN4 during astrocyte differentiation.

## Discussion

The present study describes the SeVdp-based vectors that carry the SrC switch to flexibly regulate expression of a transgene including a reprogramming factor. The SrC switch consists of two components; (1) a CRISPR RNA processing enzyme Csy4 tagged with an FKBP12-based degradation domain and (2) the target sequence Csy4RS inserted into the transgene mRNA. This system executes the regulation of the transgene entirely by addition or removal of Shield1 that attenuates the DD function. Given the minimal toxicity and superb cell permeability of Shield1 in vitro and in vivo [35, 54, 55], the system may be applicable across a broad range of cell types. In particular, the SrC switch is suitable for direct reprogramming when expression of the transgene, often a transcription factor, requires flexible and stepwise regulation throughout the course of reprogramming. The SrC switch capable of controlling a cell-specific transcription factor may be combined with a standard differentiation procedure to enhance the rate and efficiency of the differentiation process.

The SrC switch is a ligand-controlled OFF system, in which Shield1 addition diminishes degradation of DD-Csy4, which then leads to cleavage of the target mRNA. Csy4-mediated cleavage requires the 28-nt sequence Csy4RS, which can be placed at the 5′ UTR of the target mRNA with no modification whatsoever of the target protein. Even when Csy4RS is placed in frame, with addition of two extra nucleotides, after the start codon of the target mRNA, only 10 amino acids are added at the N-terminus of the target protein. This minimal modification has a negligible effect on the *L* gene function in our vector (Fig. 5). In addition, the SrC switch is a two-tiered enzymatic process (proteasome and endoribonuclease) and amplifies the dynamic range of the output. Indeed, ~ 3-fold change of DD-Csy4 results in ~ 100-fold change in EGFP fluorescence (Fig. 1). Moreover, the whole process can be reversibly controlled; for example, EGFP expression can be flexibly increased or decreased by changing the amount of Shield1 added to the cultured cells (Fig. 3). We noted that the SrC switch quickly responds to Shield1, followed by executing inhibition of target gene expression. The Nluc activity was rapidly reduced even 0.5 h after addition of Shield1 to SeV(Csy4/RS-Nluc)-infected cells, and further incubation up to 4 h strongly inhibited the Nluc expression (Fig. 2B). By contrast, we observed that EGFP expression diminished significantly only at day 5 after Shield1 treatment of SeV(Csy4/RS-EGFP)-infected cells (Fig. 1E). This difference in off-response may be attributed to distinct protein half-lives of Nluc (Nluc-PEST; 0.25 h) [56] and EGFP (26 h) [57]. Thus, the response of the SrC switch may be improved by carefully calibrating the half-life of the target protein via addition of a degron. Alternatively, incorporating multiple copies of Csy4RS, addition of mRNA degradation motifs within the target mRNA [58], or the use of nucleases such as Cse3 or CasE that are derived from CRISPRs [43] may accelerate mRNA cleavage and improve the response. We showed that the SrC switch had superior inhibitory efficacy compared to LID and AID2 systems when these systems were incorporated into SeVdp vectors. In general, SeVdp vectors produce high amounts of transgene-derived proteins in infected cells [16]. Therefore, it is possible that the ubiquitin-proteasome system cannot degrade the nascent protein tagged with LID- or mAID sufficiently when using SeVdp vectors. In contrast, the SrC switch takes advantage of DD and SeVdp vectors. A large amount of the target protein is produced in the absence of Shield1 due to the high capacity of SeVdp vectors in protein synthesis whereas the nascent DD-Csy4 is rapidly stabilized with Shield1, and then cleaves the Csy4RS-containing target mRNA continuously. This characteristic may confer a superior dynamic range to control transgene expression from SeVdp vectors.

An obvious application for the SrC switch is transcription factor-directed differentiation or direct reprogramming. Cell differentiation is governed by a set of key transcription factors that define the cell fate. These cell-type specific transcription factors are often used as a reprogramming factor to direct differentiation of ESCs or iPSCs into a specific type of cells, even without supply of extracellular signaling molecules. For example, NGN2, ISL1, and LHX3 are used for inducing motor neurons [59], MYOD1 for skeletal muscle [60], CITED2 for cardiomyocytes [61], and FOXA2 for hepatocytes [62]. Given their precise temporal and spatial patterns of expression in developing tissues, cell-type specific transcription factors should be temporally regulated to optimize directed differentiation [63, 64]. Moreover, expression levels of reprogramming factors commonly impact the efficiency of directed differentiation [65]. The SrC switch described here is well suited for such applications because of its ability to temporally and quantitatively adjust the expression level of a reprogramming factor. An exemplary case is shown in Fig. 6G. Here, once NSCs are induced from mESCs by forced expression of BRN4, reducing BRN4 expression from the vector facilitates subsequent differentiation of NSCs into astrocytes. When the exogenous BRN4 remains expressed, the astrocyte differentiation is severely compromised. Astrocytes can be induced from mESCs by embryoid body (EB) formation followed by retinoic acid treatment with similar efficiency to our SrC switch [66]. Although the current version of the SrC switch using BRN4 does not necessarily improve the astrocyte differentiation system, the SrC switch is easily adaptable for further improvement of astrocyte induction; for example, by manipulating the expression pattern of BRN4, by altering BRN4 functions via mutagenesis, or by using an alternate transcription factor.

SeVdp-based vectors have advantages as an expression vector because of their high infectivity, high copy number, persistent infection as well as absence of chromosomal integration [16, 22]. However, SeVdp-based vectors suffer from immunogenicity and, at permissive temperature (32^o^C), moderate cytotoxicity. Thus, to avert the undesirable immunogenicity and cytotoxicity, it would be best if the SeVdp vector is eliminated completely from cells when the expression of a transgene is no longer required. We have previously reported that anti-*L* gene siRNA eliminates SeVdp vectors from infected cells by blocking SeVdp replication and generates iPSCs that are completely free of the vector [22]. Anti-*L* gene siRNA depletes the L protein, a component of SeV RdRp [22], and impairs transcription and replication of the entire viral RNA. However, the successful application of anti-*L* gene siRNA owes largely to the propensity of iPSCs to form clonal colonies and proliferate rapidly, which is not necessarily the case with differentiated cells generated by direct reprogramming. To overcome this, we have engineered a three-tiered mechanism consisting of protein degradation (protease), mRNA cleavage (endoribonuclease) and genome replication (RNA polymerase) through targeting of the *L* gene by DD-Csy4 stabilized by Shield1. This three-tiered enzymatic process produces a significantly amplified output that virtually eliminates the SeVdp vector from the infected cells that neither form colonies nor proliferate rapidly. Moreover, given that Shield1 possesses superior cell permeability, the *L*-targeting SrC switch may be usable for hard-to-transfect cells, which often coincide with slowly proliferating cells.

Although the SrC switch may be applicable for RNA-based vectors, it has some limitations. First, the rate and magnitude of target gene response should be calibrated for each expressed gene, in large part, depending on the protein stability. In addition, the SrC switch is a ligand-controlled OFF switch, which is less versatile than a ligand-controlled ON switch for transient induction of a target gene. Moreover, the SrC switch based upon SeVdp vectors, which express immunogenic proteins, is more suitable for in vitro or ex vivo expression rather than in vivo expression of a gene.

## Conclusion

We have developed a ligand-controlled switch, termed SrC switch, and integrated it into SeVdp vectors. The SrC switch may be applicable to a wide range of NSRV vectors, which lack a robust gene-regulatory system for expressing a transgene. The tunable regulation of gene expression and elimination of the vector at the appropriate timing should greatly expand the applicability of vectors based on various NSRVs, for instance, in cell reprogramming [37, 38], genome editing [67], and cancer therapy [68]. Given that the SrC switch responds to Shield1 with a high signal-to-noise ratio, it may also provide a circuit system for sophisticated gene regulatory networks in synthetic biology [69]. In any event, further improvement of the SrC switch should contribute to expanding its versatility and applicability for various biological and medical applications in the future.

## Methods

### Cell culture

NIH3T3 cells were cultured in DMEM [DMEM high Glucose (Nacalai Tesque) supplemented with 10% Fetal Bovine Serum (FBS; Nissui) and 100 U/mL Penicillin/Streptomycin solution (Nacalai Tesque)]. Mouse embryonic stem cells (mESCs) EB5 were cultured in mESC medium [DMEM high Glucose supplemented with 15% FBS, 100 mM Non-Essential Amino Acids (Nacalai Tesque), 0.5 mM StemSure Monothioglycerol Solution (Wako), 1,000 U/mL Leukemia Inhibitory Factor (LIF; Wako), and 100 U/mL Penicillin/Streptomycin solution]. For NSC differentiation, mESCs were cultured in NSC medium [DMEM/F12 (Nacalai Tesque) supplemented with B27 supplement (Wako), 20 ng/mL basic-FGF (R&D system), 20 ng/mL EGF (PeproTech), 0.005% Bovine Serum Albumin (BSA; Wako), 5 µg/mL Heparin (Nacalai), 100 U/mL Penicillin/Streptomycin solution, and 50 µg/mL Ascorbic acid (Nacalai Tesque)]. NSCs were maintained in a plate coated with 5 µg/mL Laminin (Wako). For astrocyte differentiation, NSCs were cultured in astrocyte differentiation medium (DMEM/F12 supplemented with 1% FBS, B27 supplement, 0.5 mM StemSure Monothioglycerol Solution, 0.005% BSA, and 100 U/mL Penicillin/Streptomycin solution). Protein expression levels were manipulated by addition of Shield1 (TaKaRa Bio) or 5-Ph-IAA (BioAcademia).

### Production and infection of SeVdp vectors

To prepare cDNA for SeVdp vectors, cDNAs encoding human codon-optimized Csy4, aminoglycoside 3′-phosphotransferase (NeoR), EGFP, Keima-Red (KR), firefly luciferase (Luc2CP), OsTIR1(F74G), and mouse BRN4 were amplified by PCR from pLV.PC.FCRS-Z [45], pcDNA3 (Invitrogen), pEGFP-1 (TaKaRa Bio), phdKeima-Red-S1 (Medical & Biological Laboratories), pGL4.12 (Promega), pAY15 (RIKEN BRC, #RDB18334) [46], and mouse brain cDNA, respectively. cDNA encoding rat cytochrome P450 2B1 was a kind gift from Dr. Adesnik [70]. cDNA encoding Nluc-PEST was synthesized by Eurofins. To prepare the LID, the DNA fragment encoding FKBP12(F36V) with an additional mutation of C to T at the nucleotide position 320 was amplified by PCR using the pPTuner (TaKaRa Bio) as a template, followed by cloning into pUC19 to yield pUC-FKBP12(F36V). The degron sequence encoding 19 amino acids [36] was prepared by annealing of oligonucleotides, and then inserted into immediately after the 3′ end of *FKBP12(F36V)* to yield pUC-LID. To construct DD-Csy4, the DNA fragment containing the DD was amplified by PCR using the pPTuner as a template and then fused into the regions corresponding to the N- and C-termini of Csy4 using XE cocktail enzyme mix [71], followed by cloning into the pBluescript II SK (+) (Stratagene) to yield pBSK-fCsf. To construct DD-EGFP, the amplified DD sequence was incorporated into the region corresponding to the N-terminus of EGFP using XE cocktail enzyme mix. To construct DD-HA-Csy4, the DNA fragment encoding HA-tagged Csy4 with DD at its N-terminus was amplified by PCR using pBSK-fCsf as a template, and then inserted into the BglII and HindIII sites of the pPTuner. The Csy4 recognition sequence (Csy4RS), prepared by annealing of oligonucleotides, was inserted into the 5′ UTRs of the *EGFP*, *Nluc-PEST*, *KR*, *Brn4*, and SeV *L*, or after the start codon of the *L* gene using XE cocktail enzyme mix. When the 28-nt Csy4RS was inserted after the start codon, two nucleotides were added to avoid frame shift. The LID and mAID sequence were prepared by PCR using pUC-LID and pAY15 as templates, followed by incorporating into the 3′ UTR or 5′ UTR of the Nluc-PEST using XE cocktail enzyme mix, respectively. These cDNAs were used to construct cDNAs for SeV(Csy4/EGFP), SeV(Csy4/RS-EGFP), SeV(HACsy4/RS-EGFP), SeV(Csy4/RS-Nluc), SeV(Nluc-LID), SeV(OsTIR/AID-Nluc), SeV(Csy4/DD-EGFP/RS-KR), SeV(Csy4/RS-L), SeV(Csy4/P450), SeV(Csy4/P450/RS-L), SeV(Csy4/P450/ATG-RS-L), and SeV(Csy4/RS-Brn4/RS-EGFP). SeVdp vectors were prepared as described previously [22] and infected to cells at 32˚C for 14–16 h. To select vector-infected cells, 1,000 µg/mL G418 was added 2 days after infection. Oligonucleotide sequences for constructing the vectors are listed in Table S1.

### Western blotting and fluorescence detection to determine transgene expression from SeVdp vectors

DD-Csy4 expression was controlled by adding Shield1 into the culture medium. To analyze the expression of HA-tagged DD-Csy4, whole cell extracts were subjected to SDS-PAGE, and the protein level was determined by Western blotting as described previously [72] using anti-HA (Roche; 3F10; 1:5,000) and anti-α-TUBULIN (Abcam; ab7291; 1:10,000). Fluorescent protein expression was observed under AxioVision A1 (Zeiss) and quantified using a Gallios flow cytometer (Beckman Coulter). Mean fluorescence intensity was calculated using Kaluza software (Beckman Coulter).

### Luciferase assay

To measure the luciferase activity of SeV(Csy4/RS-Nluc), we seeded 6 × 10^4^ of the vector-infected NIH3T3 cells in a 48 well plate. Next day, the cells were incubated with or without Shield1 for 0.5, 1, 2, 4 h, and then lysed with Passive Lysis Buffer (Promega). To compare the luciferase activities of SeV(Csy4/RS-Nluc), SeV(Nluc-LID) and SeV(OsTIR/AID-Nluc), we seeded 6 × 10^4^ of the vector-infected NIH3T3 cells in a 48 well plate and cultured with or without Shield1 or 5-Ph-IAA. After 24 h of culture, the cells were lysed with Passive Lysis Buffer. To evaluate reversible control of SeV(Csy4/RS-Nluc), we seeded the vector-infected NIH3T3 cells and cultured with 30 nM Shield1 or dimethyl sulfoxide (DMSO) for 2 days (Day 2). Then, the cells were re-seeded and cultured without Shield1 or DMSO for 3 days (Day 5). The cells were lysed with Passive Lysis Buffer on Days 2 and 5. The same procedure was repeated and the cells were lysed on Days 7 and 10. As control, non-treated cells were lysed on Day 0. Activities of Nluc and Fluc were analyzed using the Nano-Glo Dual Luciferase Reporter Assay System (Promega) according to the manufacture’s instructions.

### Quantification of the relative copy number of the SeVdp genomic RNA

Total RNA was isolated from cells using ISOGEN (Nippon Gene), and reverse transcription was performed using Superscript III First-Strand Synthesis System (Thermo Fisher Scientific) using the primer (5′-TGGCCACTTTGTCACACTAC-3′) corresponding to SeV *NP* gene. Quantitative PCR (qPCR) analyses were performed as described previously [73]. Primers are listed in Table S2.

### Quantification of the relative number of vector-free cells

After selection with 1 mM cyclophosphamide (CPA) for 7 days, the surviving cells were incubated with Crystal violet solution [0.2% Crystal violet (Nacalai) in 2% ethanol] at 25ºC for 10 min. The stained cells were washed and then lysed by 1% SDS, and A_570_ was measured to quantify the relative cell number.

### Expression of differentiation marker genes

Immunofluorescence staining was performed as described previously [38] using anti-SSEA1 (Santa Cruz; sc-21,702; 1:250), anti-Nestin (Santa Cruz; sc-33,677; 1:250), anti-GFAP (Sigma; G9269; 1:1,000), or anti-POU3F4 (BRN4) antibody (Proteintech; 25114-1-AP; 1:500). RNA extraction, cDNA synthesis, and qPCR were performed as described above except using random primers for cDNA synthesis. Primers are listed in Table S2.

### Statistical analysis

The data are presented as the mean ± SEM or ± SD of three independent experiments. Statistical analyses were performed using a two-tailed Student’s t-test.

### Supplementary Information

**Additional file 1: Figure S1.** Infectivity of an SeVdp vector. **Figure S2.** Original images presented in Fig. 1C. **Figure S3.** Control of transgene expression derived from SeV(Csy4/RS-EGFP). **Figure S4.** Bi-directional control of transgene expression by Shield1 addition. **Figure S5.** Images of Crystal violet assay. **Figure S6.** Control of BRN4 *expression by the SrC switch.* **Table S1.** Oligonucleotide sequences for plasmid construction. **Table S2.** Primer sequences for qPCR.

## Data Availability

All data generated or analyzed during this study are included in this published article and its supplementary information files.
