# Supplementary material for: An engineered ligand-responsive Csy4 endoribonuclease controls transgene expression from Sendai virus vectors
Source: J Biol Eng. 2024 Jan 16;18:9. doi: 10.1186/s13036-024-00404-9 (PMC10790456; doi:10.1186/s13036-024-00404-9)
Supplement: Supplementary file 1 — Additional file 1: Figure S1. Infectivity of an SeVdp vector. Figure S2. Original images presented in Fig. 1C. Figure S3. Control of transgene expression derived from SeV(Csy4/RS-EGFP). Figure S4. Bi-directional control of transgene expression by Shield1 addition. Figure S5. Images of Crystal violet assay. Figure S6. Control of BRN4 expression by the SrC switch. Table S1. Oligonucleotide sequences for plasmid construction. Table S2. Primer sequences for qPCR. [file 13036_2024_404_MOESM1_ESM.pdf]

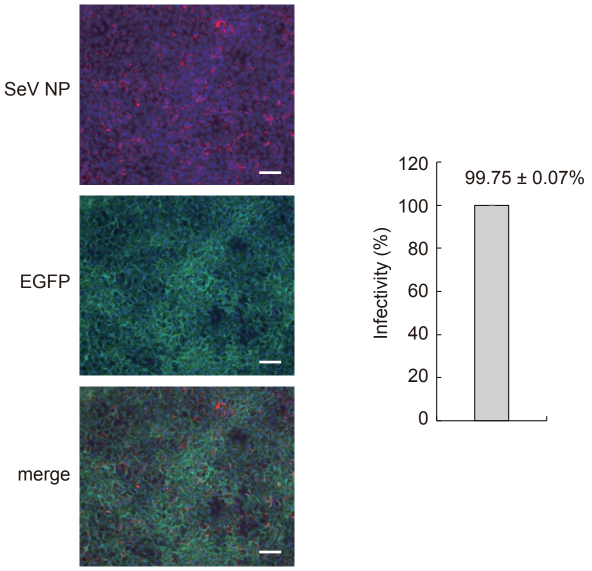

Figure S1. Infectivity of an SeVdp vector.
SeV(HACsy4/RS-EGFP) was infected to NIH3T3 cells at M.O.I. of ~5.0. SeV NP protein was immunostained 3 days after the infection. Data are represented as the means ± SEM of three independent experiments. Scale bars, 200 µm.

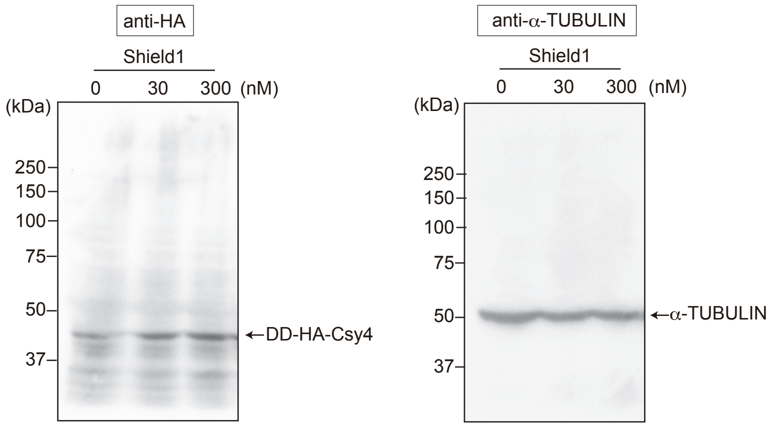

Figure S2. Original images presented in Fig. 1C.
Representitive image of three independent experiments.

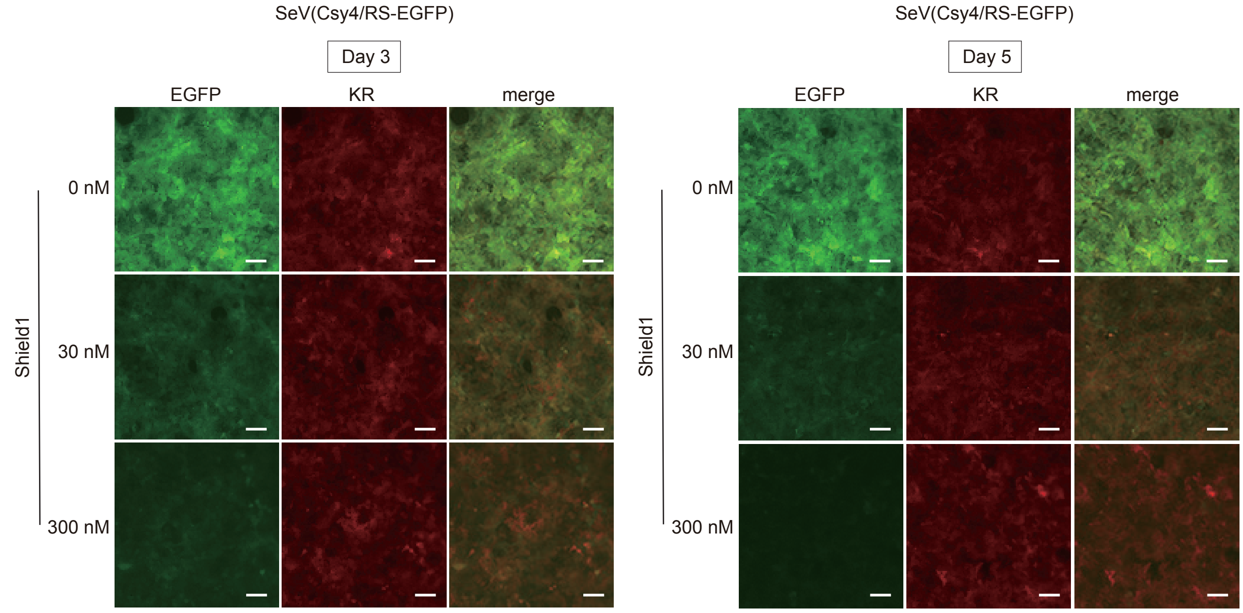

Figure S3. Control of transgene expression derived from SeV(Csy4/RS-EGFP).
SeV(Csy4/RS-EGFP)-infected NIH3T3 cells were cultured with indicated concentration of Shield1 for 3 or 5 days. EGFP and KR images were overlaid to produce merged images. Scale bars, 200 µm.

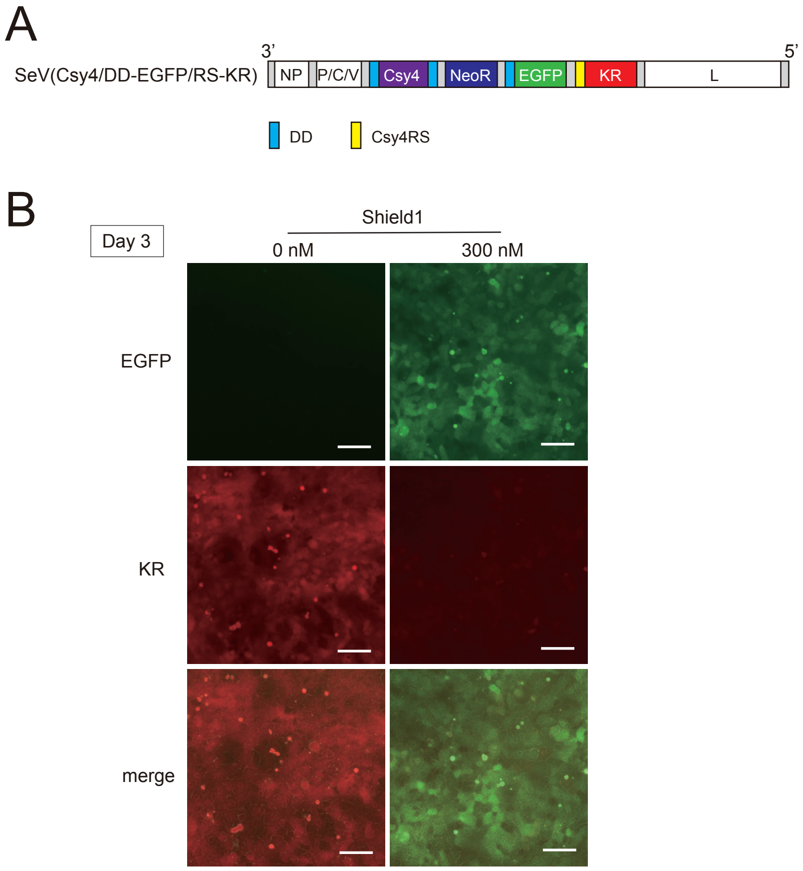

Figure S4. Bi-directional control of transgene expression by Shield1 addition.
A Structure of SeV(Csy4/DD-EGFP/RS-KR). **B** SeV(Csy4/DD-EGFP/RS-KR)-infected NIH3T3 cells were cultured with or without 300 nM of Shield1 for 3 days. EGFP and KR images were overlaid to produce merged images. Scale bars, 200 µm.

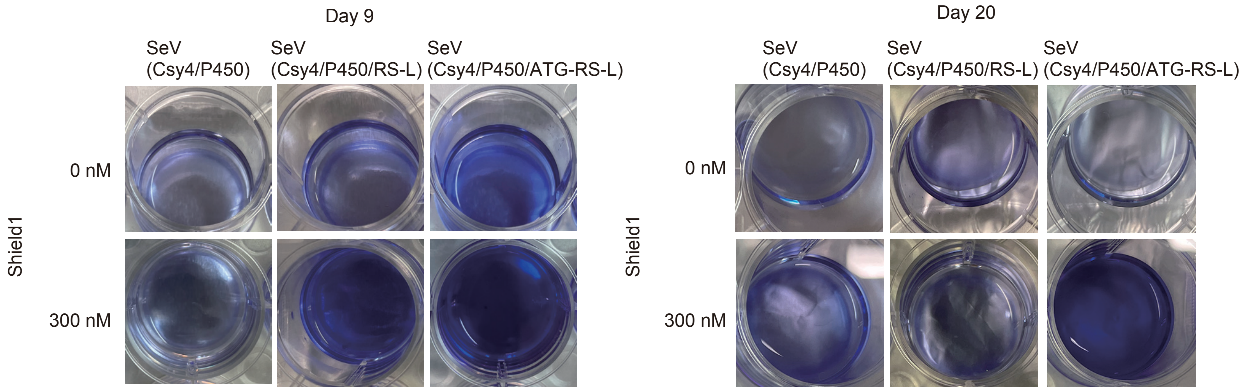

Figure S5. Images of Crystal violet assay.
Representative images of Crystal violet assay shown in Fig. 5E.

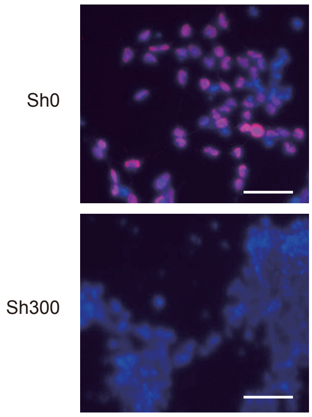

Figure S6. Control of BRN4 expression by the SrC switch.
SeV(Csy4/RS-Brn4/RS-EGFP)-infected EB5 cells were cultured with or without 300 nM Shield1, and BRN4 protein was immunostained. Scale bars, 100 μm.

Table S1. Oligonucleotide sequences for plasmid construction

cDNA amplification

Csy4	5'-CTTCTAAAACCGGAAATGGGGGACCACTATCTGGAC-3'
	5'-CACCTGCACTCCCATGAACCAAGGCACGAATCCTC-3'
NeoR	5'-GCAAGCGGCCGCTTGGCGCCGTTTCGCATGATTGAACAAG-3'
	5'-GGTCACGCGTCTCAGAAGAACTCGTCAAGAAG-3'
EGFP	5'-AGTACCTAGGATGGTGAGCAAGGGCGAG-3'
	5'-GGCGCTTAATCGACGTCTTTACTTGTACAGCTCGTCCA-3'
Keima-Red	5'-ATCGCTAGCAGCCAGCATGGTGAGCGTGATTGCCAAG-3'
	5'-GCGACCGGTCCTAAGTTTTTCTTAGGTGC-3'
Luc2CP	5'-CGGTATTTTAGCTAGCAGCCATGGAAGATGCCAAAAACATTAAGA-3'
	5'-CAAAGCCGACACCGGTCTTAGACGTTGATCCTGGCGC-3'
OsTIR1(F74G)	5'-AAATTTCACCGCTAGCACCTATGACATACTTTCCTGAAGAGGTCG-3'
	5'-AATCGACGTCACCGGTTTCACAGAATCTTCACAAAGTTGGGA-3'
BRN4	5'-ACTAGCTAGCACCTAGGGACATGGCCACAGCTGCCTCGAA-3'
	5'-GTCCGACGTCACCGGTTTCAGAGATCGTGGCAGGACGC-3'
FKBP12(F36V)	5'-AATCTCTAGAGGAGTGCAGGTGGAAACCATC-3'
	5'-ATATGGTACCACGCGTTTCCAGTTTTAGAAGCTCCACATCG-3'
DD	5'-ATGGGAGTGCAGGTGGAAAC-3'
	5'-TTCCGGTTTTAGAAGCTCCA-3'
DD-HA-Csy4	5'-CTCTAGATCTTACCCATACGATGTTCCAGATTACGCTCTTACCATGGGGGACCACTATCTGGAC-3'
	5'-CTCTAAGCTTTCATTCCGGTTTTAGAAGCTCC-3'
LID	5'-CAACGTCGACTCTAGAGGAGTGCAGGTG-3'
	5'-ACAGATCTGACGTCCCTAATTACCTCGCCGCCTTAAG-3'
mAID	5'-TTAGATCTAGCCTAGGAGCATGAAG-3'
	5'-GGGTGAACACCAGCTCCTCGCCCTTGC-3'

dsDNA production

Degron sequence of LID	5'-CGCGTGGAGTGGAGGAAGTGGCGGAGGGGGTAGTTCTCTTAAGGCGGCGAGGTAATTAGG-3'
	5'-AATTCCTAATTACCTCGCCGCCTTAAGAGAACTACCCCCTCCGCCACTTCCTCCACTCCA-3'
Csy4 recognition sequence	5'-AGTTCACTGCCGTATAGGCAGCTAAGAAAT-3'
	5'-ATTTCTTAGCTGCCTATACGGCAGTGAACT-3'

red: Csy4 RS

Table S2. Primer sequences for qPCR

SeV genomic RNA	5'-CGAAGAAGATGACGATGCC-3'
	5'-TGAGCCGATCGATGGATGAA-3'
mPax6	5'-CGCGGATCTGTGTTGCTCAT-3'
	5'-CTTAAATCCATGGCAAATCTTGTCG-3'
mSox11	5'-CACCACAGCCACAAAGAGCAA-3'
	5'-CACATGGGCACATCCAGGTT-3'
